# Evolutionary histories and mycorrhizal associations of mycoheterotrophic plants dependent on saprotrophic fungi

**DOI:** 10.1007/s10265-020-01244-6

**Published:** 2021-01-08

**Authors:** Yuki Ogura-Tsujita, Tomohisa Yukawa, Akihiko Kinoshita

**Affiliations:** 1 Faculty of Agriculture, Saga University, 1 Honjo-machi, Saga, 840-8502 Japan; 2 United Graduate School of Agricultural Sciences, Kagoshima University, 1-21-24 Korimoto, Kagoshima, 890-8580 Japan; 3 National Museum of Nature and Science, 4-1-1 Amakubo, Tsukuba, 305-0005 Japan; 4 Kyushu Research Center, Forestry and Forest Products Research Institute, Kumamoto city, Chuo-ku, Kurokami, Kumamoto 860-0862 Japan

**Keywords:** *In vitro* culture, Litter decay fungi, Orchid, Stable isotopes, Wood decay fungi

## Abstract

Mycoheterotrophic plants (MHPs) are leafless, achlorophyllous, and completely dependent on mycorrhizal fungi for their carbon supply. Mycorrhizal symbiosis is a mutualistic association with fungi that is undertaken by the majority of land plants, but mycoheterotrophy represents a breakdown of this mutualism in that plants parasitize fungi. Most MHPs are associated with fungi that are mycorrhizal with autotrophic plants, such as arbuscular mycorrhizal (AM) or ectomycorrhizal (ECM) fungi. Although these MHPs gain carbon via the common mycorrhizal network that links the surrounding autotrophic plants, some mycoheterotrophic lineages are associated with saprotrophic (SAP) fungi, which are free-living and decompose leaf litter and wood materials. Such MHPs are dependent on the forest carbon cycle, which involves the decomposition of wood debris and leaf litter, and have a unique biology and evolutionary history. MHPs associated with SAP fungi (SAP-MHPs) have to date been found only in the Orchidaceae and likely evolved independently at least nine times within that family. Phylogenetically divergent SAP Basidiomycota, mostly Agaricales but also Hymenochaetales, Polyporales, and others, are involved in mycoheterotrophy. The fungal specificity of SAP-MHPs varies from a highly specific association with a single fungal species to a broad range of interactions with multiple fungal orders. Establishment of symbiotic culture systems is indispensable for understanding the mechanisms underlying plant–fungus interactions and the conservation of MHPs. Symbiotic culture systems have been established for many SAP-MHP species as a pure culture of free-living SAP fungi is easier than that of biotrophic AM or ECM fungi. Culturable SAP-MHPs are useful research materials and will contribute to the advancement of plant science.

**Supplementary Information:**

The online version contains supplementary material available at 10.1007/s10265-020-01244-6.

## Introduction

Mycoheterotrophic plants (MHPs) are non-photosynthetic and thus completely reliant on mycorrhizal fungi for carbon uptake throughout their lifecycle (Leake 1994). Most MHPs have small vegetative organs and have an underground root/rhizome system as the main body, emerging aboveground only for reproduction. Such extreme evolution occurred independently over 40 times in all divisions of land plants, and there are ca. 580 species of MHPs (Jacquemyn and Merckx 2019). The evolution of mycoheterotrophy was accompanied by dramatic changes in a variety of characteristics, such as morphology (Leake 1994), mycorrhizal symbiosis (Ogura-Tsujita et al. 2012), pollination systems (Suetsugu 2015), seed dispersal systems (Suetsugu et al. 2015; Suetsugu 2018), and genome size and content (Barrett and Davis 2012). MHPs are therefore expected to be useful models in plant science.

**Fig. 1:**
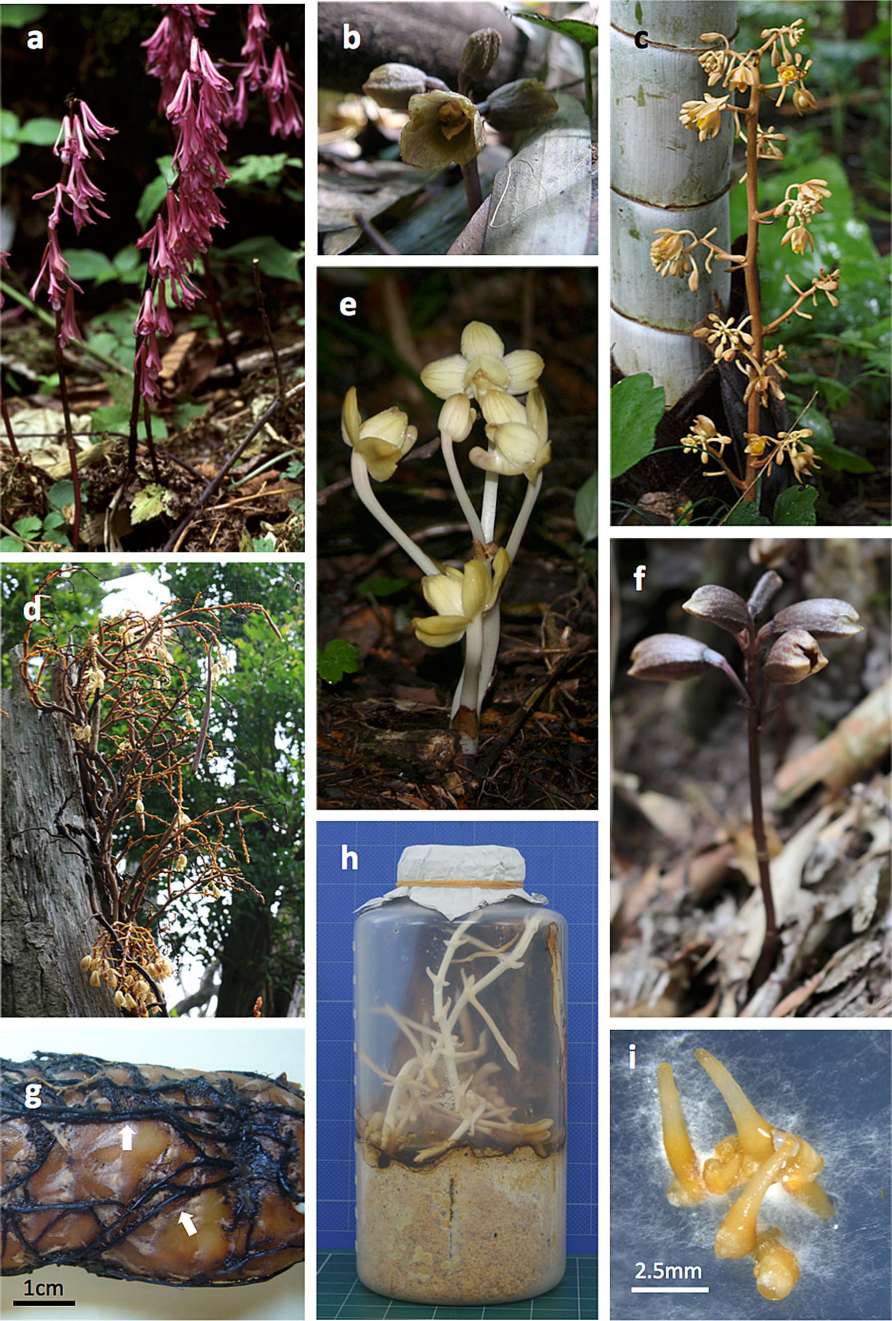
Mycoheterotrophic species associated with saprotrophic (SAP) fungi. **a** *Cremastra aphylla*, **b** *Gastrodia confusa*, **c** *Cyrtosia septentrionalis*, **d** *Erythrorchis altissima*, **e** *Yoania flava*, **f** *Gastrodia nipponica*, and **g** a tuber of *Gastrodia elata*. Black rhizomorphs are attached to the surface of the tuber. **h** *In vitro* symbiotic culture of *E. altissima* (photo by H Umata), and **i** *in vitro* symbiotic germination of *G. nipponica* seeds

Mycorrhizal symbiosis between plants and fungi is a ubiquitous type of mutualism, in which autotrophic plants exchange photosynthesized carbon for mineral nutrients obtained by mycorrhizal fungi (Smith and Read 2008). However, mycoheterotrophy represents a breakdown of this mutualism, as plants obtain carbon from fungi without photosynthesis (Merckx and Bidartondo 2008). Molecular studies for mycobiont identification have revealed two main mycorrhizal systems supporting carbon gain by MHPs; namely, via the mycorrhizal fungi of autotrophic plants —arbuscular mycorrhizal (AM) and ectomycorrhizal (ECM) fungi—and via free-living saprotrophic (SAP) fungi (Waterman et al. 2013). These two mycorrhizal systems use different carbon sources —MHPs associated with AM (AM-MHPs) and ECM fungi (ECM-MHPs) obtain carbon from surrounding autotrophic plants through shared mycorrhizal fungi, whereas MHPs associated with SAP fungi (SAP-MHPs; Fig. 1) obtain carbon from plant debris through the decomposition of wood and leaf litter. Most of the litter- or wood-decay fungi associated with SAP-MHPs are rarely found as mycorrhizal fungi in autotrophic land plants. The biology of AM- and ECM-MHPs has been reviewed by others (Bidartondo 2005; Leake 1994; Merckx 2013). However, the diversity of SAP-MHPs was elucidated only recently despite its discovery by Kusano in 1911 A critical advantage of SAP-MHPs is the feasibility of symbiotic culture. Because a pure culture of free-living SAP fungi is easier than that of biotrophic AM or ECM fungi, culture systems for several SAP-MHPs have been established (Burgeff 1936; Xu and Guo 2000; Yagame et al. 2007). These enable key questions of mycoheterotrophy to be addressed and facilitate the conservation of endangered species. Here, we review SAP-MHPs with emphasis on their evolutionary history and mycorrhizal associations. We also introduce case studies of symbiotic culture of SAP-MHPs and discuss future perspectives.

## Mycoheterotrophy

The evolution from autotrophy to mycoheterotrophy is a stepwise process involving the reduction of foliage leaves and chlorophyll content. Leafless and achlorophyllous MHPs are categorized as fully mycoheterotrophic; partial and initial mycoheterotrophy are also recognized in land plants (Merckx 2013). Partial MHPs retain normal chlorophyllous leaves and have the ability to obtain carbon from both photosynthesis and mycorrhizal fungi (Gebauer and Meyer 2003). Initial MHPs are dependent on their mycorrhizal fungi for carbon supply during the early stages of their life history and subsequently develop into autotrophic mature plants (Merckx 2013). Initial mycoheterotrophy has been observed in seed plants producing small dust-like seeds, such as in Orchidaceae and Pyroleae in Ericaceae, and also in the gametophytes of lycophytes and pteridophytes (Merckx 2013). Partial and initial mycoheterotrophy are thought to be an intermediate stage in the transition from autotrophy to mycoheterotrophy and provide insight into the evolution of the latter (Ogura-Tsujita et al. 2012).

The definitions of partial and full mycoheterotrophy are unclear. Foliage leaves are substantially reduced but still develop in some species, such as *Cephalanthera subaphylla* Miyabe and Kudô (Orchidaceae). Furthermore, some leafless MHPs have chlorophyllous reproductive shoots, indicating that photosynthesis is active during flowering and fruiting (Suetsugu et al. 2018; Zimmer et al. 2008 but also see Cameron et al. 2009). Partial and full mycoheterotrophy can be distinguished by their stable isotope signatures (Suetsugu et al. 2018; Zimmer et al. 2008; see also the section “Isotopic signature of SAP-MHPs”), but the level of mycoheterotrophy has not been evaluated for most SAP-MHPs. Therefore, in this review, species that lack foliage leaves are defined as full MHPs. Leafless species that develop chlorophyllous reproductive shoots are included as full MHPs because photosynthesis is limited to the reproductive phase. Species that have small, chlorophyllous foliage leaves are excluded from full MHP status because the leaves, the principal photosynthetic apparatus, function during the growth period. Further, leafless lianas with chlorophyllous stems such as *Pseudovanilla foliata* (F.Muell.) Garay and *Vanilla aphylla* Blume (Orchidaceae), and leafless epiphytes with chlorophyllous roots such as *Dendrophylax* and *Taeniophyllum* (Orchidaceae) are not considered MHPs because the stems or roots are photosynthetic and function throughout the life history of such species.

## Mycorrhizal symbiosis in MHPs

Three phylogenetically and physiologically distinct fungal groups are involved in mycorrhizal symbiosis in MHPs—AM, ECM, and SAP fungi (Waterman et al. 2013). AM or ECM fungi obtain carbon from their autotrophic host plants through mutualistic relationships. The AM association is the most dominant mycorrhizal symbiosis type in land plants, with more than 71% of mycorrhizal plant species associated with AM fungi (Brundrett and Tedersoo 2018). ECM fungi are mostly associated with particular tree families, such as Pinaceae and Fagaceae, and are the dominant mycorrhizal type in boreal and temperate forests (Smith and Read 2008). AM or ECM fungi are simultaneously associated with MHPs, and thus, AM- or ECM-MHPs obtain photosynthesized carbon from the surrounding autotrophic plants via shared mycorrhizal mycelia. This tripartite symbiosis allows MHPs access to the common mycorrhizal networks of AM or ECM fungi that link the surrounding autotrophic plants.

By contrast, SAP-MHPs depend on nonliving biomass. Mycoheterotrophic associations with free-living litter- or wood-decay fungi are dependent on the forest carbon cycle (Ogura-Tsujita et al. 2018; Suetsugu et al. 2020b). The decomposition of woody debris and leaf litter by SAP fungi plays a key role in regulating carbon and nutrient cycles in forest ecosystems (Berg and McClaugherty 2008). Woody debris is a major component of forest biomass, and this large carbon store represents up to 20% of the total aboveground biomass (Bradford et al. 2009; Laiho and Prescott 1999). MHPs dependent on SAP fungi can access the carbon pool via associations with litter- or wood-decay fungi, a pathway of carbon gain unique among land plants. Although carbon flow in tripartite symbiosis has been studied using stable isotopic signatures (Gebauer and Meyer 2003; Hynson et al. 2016) and labeled isotopes (Bougoure et al. 2010; McKendrick et al. 2000), carbon acquisition from plant debris in SAP-MHPs is less well understood than that in AM- and ECM-MHPs.

## Phylogeny and evolution of SAP-MHPs

**Fig. 2:**
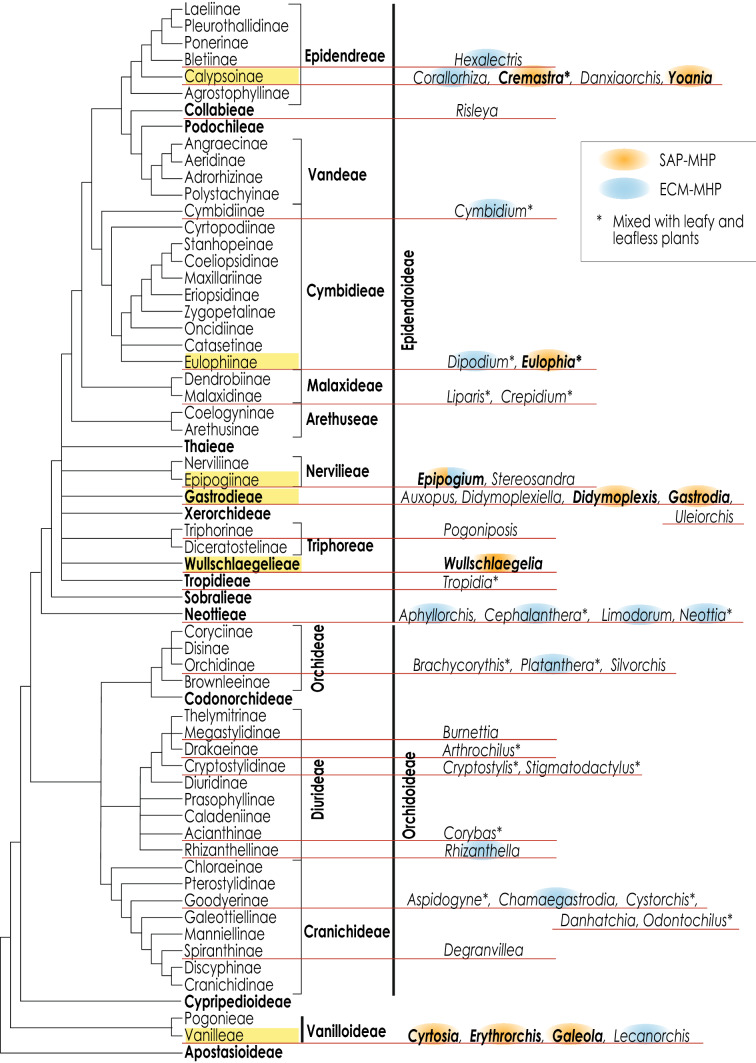
Occurrence of mycoheterotrophy within Orchidaceae. Evolutionary tracks of mycoheterotrophy are traced on a phylogenetic tree covering all major clades of the family (Chase et al. 2015). Tribes or subtribes that include saprophytic fungi-associated mycoheterotrophic plants (SAP-MHPs) are colored yellow. Genera that include SAP or ectomycorrhizal (ECM) fungi-associated MHPs (ECM-MHPs) are colored red and blue, respectively. The genus *Epipogium* comprises both SAP- and ECM-associated species. Genera of unknown mycorrhizal status are not colored. Asterisks indicate genera that include both leafy and leafless species

Among the MHPs for which the mycorrhizal fungi have been surveyed, SAP-MHPs include 28 species from 10 genera, all belonging to Orchidaceae (Fig. 2; Table 1). The evolutionary tracks of mycoheterotrophy within Orchidaceae were traced in a phylogenetic tree covering all of the major clades of the family (Chase et al. 2015; Fig. 2). It is likely that full mycoheterotrophy evolved independently at least 41 times, and that SAP-associated mycoheterotrophy (SAP-MH) evolved at least nine times within Orchidaceae (Fig. 2). When a group includes only SAP-MHPs, SAP-MH evolved in their common ancestor, whereas when a group comprises a mixture of SAP-MHPs and species associated with other mycorrhizal types, SAP-MH evolved in that clade. SAP-MHPs are found in the second-basal-most subfamily Vanilloideae and five tribes of the latest diverged subfamily Epidendroideae, showing that SAP-MH evolved in various lineages in the family.

**Table 1:** Mycoheterotrophic species associated with litter- or wood-decaying fungi, and the taxonomic affiliations of their mycobionts

Plant taxa	Taxonomic affiliation of mycobionts		Analyses^a^	References	Notes
	Order	Taxa			
*Cremastra aphylla*	Agaricales	*Coprinellus domesticus, Coprinellus* sp.	Molecular identification, sporocarp formation	Yagame et al. (2018)	One of the isolates was identified as *C. domesticus.*
*Cyrtosia javanica*	Polyporales	Meripilaceae	Molecular identification, stable isotopes	Lee et al. (2015)	The Meripilaceae fungi were identified as *Physisporinus* by Yamashita et al. (2020).
*C. septentrionalis* (*Galeola septentrionalis*)^b^	Agaricales	*Armillaria mellea*	Rhizomorph morphology, isolate characteristics	Hamada (1939, 1940)	
		*Armillaria mellea*	Symbiotic culture	Terashita (1985)	Aseptic seedlings formed mycorrhizae with *A. mellea*.
		*Armillaria tabescens*	Sporocarp formation	Terashita and Chuman (1987)	
		*Armillaria borealis, A. cepistipes, A. gallica* (*A. bulbosa*), *A. mellea, A. tabescens*	SI test	Terashita and Chuman (1989), Terashita (1996)	Possibly *A. borealis*, but further identification is required.
		*Armillaria*	Isozyme	Matsushita et al. (1996)	The fungal isolates were assigned to four biological species.
		*Armirallia jezoensis*	SI test	Cha and Igarashi (1996)	
		*Armirallia jezoensis*	PCR-RFLP	Terashima et al. (1998)	
		*Armillaria mellea*	SI test, RAPD	Ota et al. (2000)	
		*Armillaria gallica, A. mellea, A. tabescens*	Symbiotic culture	Umata et al. (2013)	Seed germination was stimulated, but no further growth was observed.
	Polyporales	Meripilaceae	Symbiotic culture, molecular identification	Umata et al. (2013)	Seed germination and following seedling growth were promoted. The Meripilaceae fungus was identified as *Physisporinus* by Yamashita et al. (2020).
	Russulales	*Xylobolus annosus*	Symbiotic culture	Umata et al. (2013)	Seed germination was stimulated, but no further growth was observed.
	Cantharellales	*Rhizoctonia repens*	Symbiotic culture	Masuhara and Katsuya (1991)	Aseptic seedlings formed mycorrhizae with *R. repens.*
	–	–	–	Nakamura et al. (1975), Nakamura (1982)	The aseptic seed germination was observed.
	–	–	–	Umata et al. (2006)	No fungal peloton was observed in the protocorms obtained from *in situ* seed germination.
	–	–	Stable isotopes	Motomura et al. (2010)	
	–	–	Radiocarbon	Suetsugu et al. (2020b)	
*Didymoplexis micradenia* (*D. minor*) ^b^	Agaricales	*Marasmius coniatus* var. *didymoplexis*	Symbiotic culture, sporocarp formation	Burgeff (1932, 1936, 1959)	
*D. pallens*	Agaricales	*Marasmius coniatus* var. *didymoplexis*	Symbiotic culture, sporocarp formation	Burgeff (1932, 1936, 1959)	
	–	–	–	Irawati (2002)	Aseptic seedlings produced inflorescences.
*Epipogium roseum*	Agaricales	*Coprinellus* (*Coprinus*)^b^, *Psathyrella*	Molecular identification	Yamato et al. (2005)	
		*Coprinellus*	Molecular identification, symbiotic culture	Yagame et al. (2007)	
		*Coprinellus disseminatus*	Sporocarp formation	Yagame et al. (2008)	
*Erythrorchis altissima* (*Galeola altissima, E. ochobiensis*) ^b^	Hymenochaetales	*Erythromyces crocicreas* (*Hymenochaete crociceras*)^b^	Isolate characteristics	Hamada and Nakamura (1963)	
	Agaricales	*Lentinula edodes*	Symbiotic culture	Umata (1998a)	See also supplemental information of Ogura-Tsujita et al. (2018) for a series of studies by Umata.
		*Lyophyllum shimeji*	Symbiotic culture	Umata (1997a)	Seed germination was stimulated, but no promotive effect for further development.
		*Pleurotus ostreatus*	Symbiotic culture	Umata et al. (2000a)	
		*Gymnopus*, *Hypholoma*, *Mycena*, *Neonothopanus*	Molecular identification	Ogura-Tsujita et al. (2018)	The *Gymnopus* sequence was nested within the *Marasmiellus* clade (Fig. S2).
	Atheliales	*Athelia*	Molecular identification	Ogura-Tsujita et al. (2018)	
	Auriculariales	*Auricularia polytricha*	Symbiotic culture	Umata (1997b)	
	Cantharellales	*Ceratobasidium*, *Tulasnella*	Molecular identification	Ogura-Tsujita et al. (2018)	
	Corticiales	*Vuilleminia*	Molecular identification	Ogura-Tsujita et al. (2018)	
	Hymenochaetales	*Erythromyces crocicreas*	Symbiotic culture	Umata (1995, 1998b)	
		*Phellinus* sp.	Symbiotic culture	Umata (1995, 1998b)	
		*Phellinus gilvus*, *Phellinus wahlbergii*	Symbiotic culture	Umata et al. (2000a)	
		*Fuscoporia*, Hymenocaetaceae	Molecular identification	Ogura-Tsujita et al. (2018)	
	Polyporales	*Fomitopsis vinosa*, *Lentinus sajor-caju*, *Panus tigrinus*	Symbiotic culture	Umata et al. (2000a)	
		*Ganoderma australe*, *Loweporus tephroporus*, *Microporus affinis*	Symbiotic culture	Umata (1995, 1998b)	
		*Lenzites betulinus*, *Trametes hirsuta*	Symbiotic culture	Umata (1999)	
		*Ceriporia*, *Hyphoderma*, *Ischnoderma, Microporus*, *Phanerochaete*, Phanerocaetaceae, *Phlebia*, *Phlebiopsis*, *Stereum*	Molecular identification	Ogura-Tsujita et al. (2018)	
	Russulales	*Hericium erinaceus*, *Xylobolus annosus*	Symbiotic culture	Umata et al. (2000a)	
		*Asterostroma*, *Coniophorafomes matsuzawae*, *Russula*^c^, *Scytinostroma*	Molecular identification	Ogura-Tsujita et al. (2018)	
	Sebacinales Russula	Serendipitaceae	Molecular identification	Ogura-Tsujita et al. (2018)	
	Trechisporales	*Hyphodontia*, *Sitstostremastrum*, *Trechispora*, Trechisporales, *Trichaptum* cf. *durum*	Molecular identification, stable isotopes	Ogura-Tsujita et al. (2018)	
*Erythrorchis cassythoides*	Agaricales, Russulales	*Russula*^c^, *Gymnopus*	Molecular identification	Dearnaley (2006)	The *Gymnopus* sequence was nested within the *Marasmiellus* clade (Fig. S2).
*Eulophia zollingeri*	Agaricales	*Psathyrella* cf. *candolleana*	Molecular identification	Ogura-Tsujita and Yukawa (2008)	
	–	–	Radiocarbon	Suetsugu et al. (2020b)	
*Galeola falconeri*	Polyporales	Meripilaceae	Molecular identification, stable isotopes	Lee et al. (2015)	The Meripilaceae fungus was identified as *Physisporinus* by Yamashita et al. (2020).
*G. nudifolia* (*G. hydra*)^b^	Polyporales	*Fomes*		Burgeff (1959)	
*Gastrodia appendiculata*	Agaricales	*Mycena*	Molecular identification, stable isotopes	Lee et al. (2015)	
*G. callosa*	–	Mycelia without clamp connection	Microscopic observation	Burgeff (1932, 1959)	
*G. confusa*	Agaricales	*Clitocybula, Gymnopus, Mycena*	Molecular identification, stable isotopes	Ogura-Tsujita et al. (2009)	*Mycena* was the most dominant. The sequences of *Clitocybula* and *Gymnopus* were nested within the hydropoid and *Marasmiellus* clades, respectively (Fig. S2).
		*Mycena*	Molecular identification, symbiotic culture	Shimaoka et al. (2017)	
	Cantharellales	*Ceratobasidium*	Molecular identification	Ogura-Tsujita et al. (2009)	
*G. cunninghamii*	Agaricales	*Armillaria mellea*	Morphology of rhizomorph	Campbell (1962)	Rhizomorphs were attached to the tuber surfaces.
*G. elata*	Agaricales	*Armillaria mellea*	Rhizomorph morphology	Kusano (1911)	See also the review by Xu and Guo (2000) and Liu et al. (2010) for *G. elata* study.
		*A. gallica*	SI test	Mohammed et al. (1994)	
		*A. gallica, A. jezoensis, A. ostoyae, A. sinapina, A. singula*	SI test, isozyme	Cha and Igarashi (1995)	
		*A. gallica*	SI test, sporocarp formation	Kikuchi et al. (2008b)	
		*A. cepistipes, A. gallica, A. nabsnona*	SI test	Kikuchi et al. (2008a)	
		*A. nabsnona*	SI test, molecular identification	Sekizaki et al. (2008)	
		*Armirallia* (seven lineages)	Molecular identification	Guo et al. (2016)	
		*Armirallia*	Molecular identification, symbiotic culture	Yeh et al. (2017)	
		*Mycena anoectochila*	Symbiotic culture	Guo et al. (1997)	
		*Mycena dendrobii*	Symbiotic culture	Guo et al. (1999), Pan et al. (2015)	
		*Mycena orchidicola*	Symbiotic culture	Fan et al. (1996)	
		*Mycena osmundicola, Mycena*	Symbiotic culture	Hong et al. (2002)	
		*Mycena osmundicola*	Sporocarp formation, symbiotic culture	Xu and Guo (1989)	
		*Mycena osmundicola*	Symbiotic culture	Kim et al. (2006)	
		*Armillaria mellea, Mycena osmundicola*	Symbiotic culture	Park et al. (2012)	
		*Mycena*	Molecular identification, symbiotic culture	Park and Lee (2013a)	
		*Armillaria mellea, Mycena*	Symbiotic culture	Park and Lee (2013b)	
		*Mycena*	Symbiotic culture, TEM	Li et al. (2020)	
	Agaricales and others	Unidentified Agaricales and others	Illumina sequencing	Chen et al. (2019)	Identified from seedlings.
	Hymenochaetales	*Resinicium*	Illumina sequencing	Chen et al. (2019)	Identified from seedlings.
	–	–	Radiocarbon	Suetsugu et al. (2020b)	
*G. flabilabella*	Agaricales	*Hydropus*	Molecular identification, stable isotopes	Lee et al. (2015)	
	Agaricales and others	*Mycena* and others	Illumina sequencing	Liu et al. (2015)	
*G. fontinalis*	Agaricales	*Gymnopus, Mycena*	Molecular identification, stable isotopes	Lee et al. (2015)	
*G. javanica*	Agaricales	*Xerotus javanicus*	Sporocarp formation	Burgeff (1936, 1959)	
*G. lacista*	–	–	Stable isotopes	Sommer et al. (2012)	
*G. minor*	–	Clamp bearing fungus	Isolate characteristics	Campbell (1963)	
*G. nantoensis*	Agaricales	*Mycena*	Molecular identification, stable isotopes	Lee et al. (2015)	
*G. nipponica*	Agaricales	*Crinipellis, Clitocybula, Gymnopus, Marasmiellus, Marasmius, Mycena*	Molecular identification	Kinoshita et al. (2016)	The sequences of *Crinipellis, Critocybula, Gymnopus, Marasmiellus* and *Marasmius* were spread into the Omphalotaceae, Marasmiaceae and hydropoid clades (Fig. S2).
	Auriculariales	*Auricularia*	Molecular identification	Kinoshita et al. (2016)	
	Corticiales	*Corticium*	Molecular identification, symbiotic culture	Shimaoka et al. (2017)	
	Hymenochaetales	*Resinicium*	Molecular identification	Kinoshita et al. (2016)	
	Polyporales	Meruliaceae, *Phlebiopsis*, Polyporales	Molecular identification	Kinoshita et al. (2016)	
		*Theleporus*	Molecular identification, symbiotic culture	Shimaoka et al. (2017)	
	Russulales	*Lactarius*^c^, Peniophoraceae, *Russula*^c^	Molecular identification	Kinoshita et al. (2016)	
	Sebacinales	*Sebacina*^c^	Molecular identification	Kinoshita et al. (2016)	
	Trechisporales	*Trechispora*	Molecular identification, symbiotic culture	Shimaoka et al. (2017)	
*G. pubilabiata*	Agaricales	*Crinipellis, Clitocybula, Gymnopus, Marasmiellus, Marasmius, Mycena*, Pterulaceae	Molecular identification	Kinoshita et al. (2016)	The sequences of *Crinipellis, Clitocybula, Gymnopus, Marasmiellus* and *Marasmius* were spread into the Omphalotaceae, Marasmiaceae and hydropoid clades (Fig. S2).
		*Mycena*	Molecular identification, symbiotic culture	Higaki et al. (2017)	
	Cantharellales	*Tulasnella*	Molecular identification	Kinoshita et al. (2016)	
	Polyporales	*Diplomitoporus rimosus*	Molecular identification	Kinoshita et al. (2016)	Fungal ITS sequences had 100% similarity with *D. rimosus*.
		*Diplomitoporus rimosus*	Molecular identification, symbiotic culture	Shimaoka et al. (2017)	Fungal ITS sequence from protocorm shared 558/559 bp identity with that from *D. rimosus*.
	–	Isolates from *G. confusa* (*G. verrucosa)*	Symbiotic culture	Umata et al. (2000b)	
*G. sesamoides*	Polyporales	Probably *Fomes mastoporus*	Field observation	Campbell (1964)	Mycelium, that was similar to the mycobiont of *G. sesamoides*, was traced to the sporocarp of *F. mastoporus*.
	Agaricales	*Campanella, Marasmius*	Molecular identification, stable isotopes	Dearnaley and Bougoure (2010)	The sequences of *Campanella* and *Marasmius* were nested within the campanelloids and Omphalotaceae clades, respectively (Fig. S2).
	–	Clamp bearing fungus	Microscopic observation	McLennan (1959)	
	–	–	Stable isotopes	Gomes et al. (2020)	
*G. similis*	Hymenochaetales	*Resinicium, Mycena, Gymnopus*	Molecular identification, stable isotopes	Martos et al. (2009)	*Resinicium* is the most dominant. The *Gymnopus* sequence was nested within the *Marasmiellus* clade (Fig. S2).
*G. verrucosa*	–	Clamp bearing isolates from *G. nipponica* and *G. verrucosa*	Symbiotic culture	Tashima et al. (1978)	Plant identification is erroneous and may represent either *Gastrodia confusa* or *G. pubilabiata* (H. Umata, personal communication).
*Wullschlaegelia calcarata*	Agaricales	*Gymnopus, Mycena*	Molecular identification	Martos et al. (2009)	A species was wrongly identified as *W. aphylla* in Martos et al. (2009) (see Hatté et al. 2020). The *Gymnopus* sequences were nested within the two clades of Omphalotaceae (Fig. S2).
			Radiocarbon	Hatté et al. (2020)	
*Yoania amagiensis*	Polyporales	*Physisporinus* (four OTUs)	Molecular identification	Yamashita et al. (2020)	
*Y. flava*	–	Unidentified isolate from *Y. flava* rhizome	Symbiotic culture	Tsuda et al. (2004)	Asymbiotic culture was also achieved.
	Polyporales	*Physisporinus* (a single OTU), Thelephoraceae^c^	Molecular identification	Yamashita et al. (2020)	A single *Physisporinus* OTU is dominantly detected.
*Y. japonica*	Polyporales	*Physisporinus* (two OTUs)	Molecular identification	Yamashita et al. (2020)	A single OTU is dominantly detected.
	–	–	Radiocarbon	Suetsugu et al. (2020b)	

^a^Methods for fungal identification are shown. Molecular identification: Identification by using extract DNA from the fungal isolates or mycorrhizal roots. Sporocarp formation: Identification by morphology of sporocarp formed from fungal isolates. Isolate characteristics: Fungal isolates were identified by morphological and cultural characteristics. SI test: Somatic incompatibility test of fungal isolates. Symbiotic culture: Plants were co-cultured with fungi. Illumina sequencing: Fungal community in mycorrhizal tissue was assessed by llumina sequencing. TEM: Transmission electron microscopy. Stable isotope analysis and radiocarbon approach are also indicated as “Stable isotopes” and “Radiocarbon”, respectively

^b^Binomials in parentheses are those used in the original publications. Current taxonomic literature suggests that these names are appropriate to treat as synonyms, ^c^ Ectomycorrhizal fungi

Mycoheterotrophy dependent on SAP fungi probably evolved twice or more in Vanilloideae, which encompasses three SAP-MH genera—*Galeola*, *Cyrtosia*, and *Erythrorchis*. Cameron et al. (2009) and Cameron (2011) showed that the paired genera *Galeola*-*Cyrtosia* and *Erythrorchis*-*Pseudovanilla* form a clade (Fig. S1). *Pseudovanilla* is the only chlorophyllous genus in this clade. There are two alternative hypotheses on the evolution of mycoheterotrophy in this clade. One is that mycoheterotrophy evolved twice from the common ancestor of *Galeola*-*Cyrtosia* and the ancestor of *Erythrorchis*. The other is that mycoheterotrophy evolved once from the common ancestor of the four genera and was subsequently reversed in *Pseudovanilla*, a photosynthetic plant. The latter is implausible because evolutionary processes to achieve full mycoheterotrophy cause the loss of multiple genes regulating photosynthesis (Delannoy et al. 2011; Li et al. 2020), and reversal from full mycoheterotrophy to autotrophy requires the reorganization of these functional genes. Thus, SAP-MHPs likely evolved at least twice in Vanilloideae.

In Epidendroideae, SAP-MH likely evolved at least seven times, viz., *Wullschlaegelia*, *Gastrodia*, *Didymoplexis*, *Epipogium roseum* (D.Don) Lindl., *Eulophia zollingeri* (Rchb.f.) J.J.Sm., *Cremastra aphylla* T.Yukawa, and *Yoania* (Fig. 2). *Wullschlaegelia* comprises two mycoheterotrophic species and *W. calcarata* Benth. is recognized as an SAP-MHP (Hatté et al. 2020; Martos et al. 2009). *Gastrodia* is the largest genus of SAP-MHPs and includes ca. 100 species (WCSP 2020), among which 13 have been reported to be SAP-MHPs (Table 1). *Didymoplexis* comprises 20 species, two of which—*D. micradenia* Hemsl. (synonym, *D. minor* J.J.Sm.) and *D. pallens* Griff.—exhibit SAP-MH (Burgeff 1932, 1936). The mycoheterotrophic genus *Epipogium* comprises both SAP- and ECM-associated species. *Epipogium roseum* was reported to be an SAP-MHP (Yamato et al. 2005). *Eulophia* and *Cremastra* contain both leafy and leafless species, and SAP-MH was reported for both *Eulophia zollingeri* (Ogura-Tsujita and Yukawa 2008; Suetsugu et al. 2020b) and *C. aphyllla* (Yagame et al. 2018). The mycoheterotrophic genus *Yoania* includes four species, three of which are SAP-MHPs (Suetsugu et al. 2020b; Yamashita et al. 2020). In *Cremastra* and *Epipogium*, speciation stopped occurring subsequent to SAP-MH evolution. By contrast, SAP-MH did not lead to an evolutionary dead end in *Gastordia*, and this genus was likely diversified by the establishment of novel symbioses with various SAP fungi (Kinoshita et al. 2016).

## Diversity of mycobionts in SAP-MHPs

Mycobionts of SAP-MHPs include leaf-litter or wood basidiomycete fungi (Table 1). Leaf-litter-decaying fungi colonize the topsoil and decompose plant leaf litter and other soil organic matter (Osono 2007), whereas wood-decaying fungi inhabit living trees, the trunks of standing dead trees, stumps, or fallen logs and degrade wood lignocellulose (Stokland et al. 2012). The activities of enzymes that catalyze the degradation of natural polymers, including lignin and plant cell-wall polysaccharides (mainly cellulose and hemicellulose), in both fungal groups are higher than those in AM and ECM fungi (Kohler et al. 2015). Because of their saprotrophic nature, mycobionts of MHPs isolated from plant roots are amenable to pure culture, facilitating the identification of fungal species. Fungal isolates often develop basidiocarps on culture medium and can be identified morphologically (Burgeff 1932; Kikuchi et al. 2008a; Terashita and Chuman 1987; Xu and Guo 2000; Yagame et al. 2008, 2018).

Most leaf-litter-decaying fungi that associate with MHPs belong to families in Agaricales, such as Mycenaceae, Marasmiaceae, and Omphalotaceae, which are the main fungal partners of *Gastrodia* and *Didymoplexis* (Table 1). These fungi are found worldwide as common saprobes in decaying plant materials (Kirk et al. 2008), but their taxonomic status is controversial (Wilson and Desjardin 2005). We conducted a phylogenetic analysis using published internal transcribed spacer (ITS) sequences of mycobionts of SAP-MHPs, including fungi molecularly identified as *Marasmius*, *Marasmiellus*, *Gymnopus*, *Clitocybula*, *Crinipellis*, *Campanella*, and *Hydropus* (Fig. S2). Recent studies that updated the phylogenetic placement of these fungal lineages (Antonín et al. 2019; Oliveira et al. 2019; Sandoval-Leiva et al. 2016) were employed as references. The sequences mostly clustered in Marasmiaceae and Omphalotaceae, with some in the hydropoid clade, which mainly includes wood-decaying fungi (Antonín et al. 2019). Most of the sequences clustered in the *Marasmiellus* clade, and the mycobionts of *Gastrodia similis* Bosser, *Gastrodia pubilabiata* Sawa, and *Gastrodia confusa* Honda and Tuyama were closely related with 100% bootstrap support (BS). Furthermore, mycobionts of *G. pubilabiata* and *Gastrodia nipponica* (Honda) Tuyama were closely related within the *Marasmiellus*, campanelloid, and *Porotheleum* clades (99–100% BS). These results indicate that particular fungal lineages are associated with SAP-MHPs, although a variety of fungal species participate in mycoheterotrophy. Members of *Mycena* are the most common mycobionts of *Gastrodia* species (Table 1). Although *Mycena* species are pure saprophytes, recent *in vitro* investigations revealed that several *Mycena* species have saprotrophic and biotrophic abilities (Thoen et al. 2020). These species can penetrate tree roots, and one *Mycena* species facilitated nutrient transfer to the plant. Interestingly, mycobionts of *G. pubilabiata* and *G. nipponica* detected by Kinoshita et al. (2016) exhibit high sequence similarity (> 98%) with three of these *Mycena* species— *Mycena galopus* (Pers.) P.Kumm., *Mycena albidolilacea* Kühner and Maire, and *Mycena olivaceomarginata* (Massee) Massee. Therefore, *Mycena* species that associate with SAP-MHPs could have biotrophic potential, and further evaluation of their trophic mode is warranted.

The wood-decaying fungi that associate with SAP-MHPs are predominantly members of Agaricales (Basidiomycota), such as *Armillaria* (Physalacriaceae), *Psathyrella* (Psathyrellaceae), and *Coprinellus* (Psathyrellaceae) (Table 1). *Armillaria* species are the main symbionts of *Gastrodia elata* Blume (Kusano 1911) and *Cyrtosia septentrionalis* (Rchb.f.) Garay (Fig. 1c; Hamada 1939). Mycobionts from *G. elata* and *C. septentrionalis* formed sporocarps and were identified as *Armillaria gallica* Marxm. & Romagn. (Kikuchi et al. 2008a) and *Armillaria tabescens* (Scop.) Emel (Terashita and Chuman 1987), respectively. *Psathyrella* and *Coprinellus*, both belonging to the Psathyrellaceae, were found in *Epipogium roseum* and *Eulophia zollingeri* (Ogura-Tsujita and Yukawa 2008; Yamato et al. 2005). Mycobionts of *E. roseum* and *Cremastra aphylla* (Fig. 1a) were identified as *Coprinellus disseminatus* (Pers.) J.E. Lange (Yagame et al. 2008) and *Coprinellus domesticus* (Bolton) Vilgalys, Hopple & Jacq. Johnson (Yagame et al. 2018), respectively, by sporocarp morphology and molecular identification. Besides Agaricales fungi, SAP symbionts include wood-decay fungi from other Basidiomycota orders. *Gastrodia similis*, a tropical MHP, associates with *Resinicium* of the Hymenocaetales (Martos et al. 2009). Wood-decay basidiomycetes of the Trechisporales, Polyporales, Corticiales, Russulales, and Atheriales were found in roots of a climbing MH orchid, *Erythrorchis altissima* (Blume) Blume (Fig. 1d; Ogura-Tsujita et al. 2018). Symbioses between multiple species of wood-decay fungi and *E. altissima* were confirmed by *in vitro* symbiotic culture by Umata (1995, [Bibr CR141][Bibr CR142], [Bibr CR143][Bibr CR144], 1999; Table 1). Further, Yamashita et al. (2020) found *Physisporinus* (Meripilaceae, Polyporales) as a fungal partner of SAP-MHPs. This genus is predominantly associated with *Yoania* species (Fig. 1e) as well as two other SAP-MH genera, *Cyrtosia* and *Galeola*. Although the litter-decay fungi found in SAP-MHPs comprise three Agaricales families; i.e., Mycenaceae, Marasmiaceae, and Omphalotaceae, highly divergent wood-decay fungal families are involved in SAP-MH associations.

A wood-decaying fungus, *Armillaria mellea* (Vahl) P. Kumm. sl, was reported to be a symbiont of SAP-MHPs by Kusano (1911) and Hamada (1939). This fungus is one of the largest and longest-lived terrestrial organisms and has been reported to cover an area of up to 965 ha with an age of up to ca. 8650 years (Ferguson et al. 2003). Therefore, associating with *A. mellea* sl allows MHPs access to the huge carbon pool in forests. This fungus has been recognized as a species complex (Korhonen 1978), and seven *Armillaria* species associated with Japanese *Gastrodia elata* have been recognized for *A. mellea* sl (Cha and Igarashi 1995; Kikuchi et al. 2008b). At least seven *Armillaria* lineages are associated with Chinese *G. elata* (Guo et al. 2016). Although *A. mellea* sl includes pathogens that cause root-rot disease in woody plants, *Armillaria gallica*, a major symbiont of *G. elata*, is a weak pathogen that inhabits decayed wood and litter (Mohammed et al. 1994). Rhizomorphs, i.e., linear mycelial organs, are well-developed in *A. mellea* sl (Roll-Hansen 1985) and are often attached to the tuber surface of *G. elata* (Fig. 1g; Kusano 1911). Such rhizomorphs can be traced from plant tubers or roots to decayed wood (Cha and Igarashi 1996; Kikuchi et al. 2008b). *A. mellea* rhizomorphs transport water and phosphate efficiently (Cairney et al. 1988), indicating that fungal rhizomorphs play an important role in nutrient transport between MHPs and rhizomorph-forming mycobionts.

ECM fungi occasionally associate with SAP-MHPs although dominant fungal partners are SAP fungi (more than half of total abundance). In such cases, SAP-MHPs are simultaneously associated with both ECM and SAP fungi. ECM *Russula* fungi were found to associate with *Erythrorchis cassythoides* (A.Cunn. ex Lindl.) Garay (Dearnaley 2006) and *Erythrorchis altissima* (Ogura-Tsujita et al. 2018). Mycobionts of *Gastrodia nipponica* (Fig. 1f) included ECM fungi in Russulaceae (8.6% in frequency) and Sebacinaceae (6.2%) as well as SAP fungi (Kinoshita et al. 2016). Because those ECM fungi are the main symbionts in ECM-MHPs (Ogura-Tsujita et al. 2012; Selosse et al. 2002; Taylor and Bruns 1999), they could also be symbiotic with SAP-MHPs although they are not main symbionts.

Most orchids are associated with so-called rhizoctonia fungi, including those in the basidiomycete families Tulasnellaceae, Ceratobasidiaceae, and Serendipitaceae (Rasmussen 2002; Yukawa et al. 2009). Associations with rhizoctonia fungi are occasionally observed in SAP-MHPs, such as *Erythrorchis altissima* (Ogura-Tsujita et al. 2018) and *Gastrodia* species (Kinoshita et al. 2016). Rhizoctonia fungi exhibit divergent trophic strategies; they can be plant pathogens, endophytes, saprophytes, orchid mycorrhizal or ectomycorrhizal fungi (Roberts 1999; Veldre et al. 2013). However, rhizoctonia fungi isolated from leafy orchid roots are saprophytes and are thus able to obtain nutrients from plant debris (Rasmussen 1995). These fungi are involved in initial or partial mycoheterotrophy in leafy Orchidaceae species (Schiebold et al. 2018; Stöckel et al. 2014). Although rhizoctonia fungi are involved in mycoheterotrophy with the albino forms of usually chlorophyllous orchid species (Suetsugu et al. 2019), saprophytic rhizoctonia fungi are only occasionally associated with fully mycoheterotrophic species. This suggests that rhizoctonia fungi possess insufficient physiological functionality to support the growth of full MHPs (Martos et al. 2009).

## **Specificity of mycobionts**

Most land plants have generalized associations with AM or ECM fungi (Smith and Read 2008), but AM-MHPs (Gomes et al. 2017; Merckx and Bidartondo 2008; Yamato et al. 2011) and ECM-MHPs (Ogura-Tsujita et al. 2012; Selosse et al. 2002; Taylor and Bruns 1999) typically have highly specific associations with a narrow phylogenetic range of fungi. In SAP-MHPs, the specificity varies among plant species, from a highly specific association with a single fungal species to broad interactions with multiple fungal orders. Individuals of the SAP-MHP *Eulophia zollingeri* from seven populations in Japan, Taiwan, and Myanmar associate exclusively with *Psathyrella candolleana* (Fr.) Maire sl (Ogura-Tsujita and Yukawa 2008). Most mycobionts of *Gastrodia confusa* from 10 populations separated by 5–1000 km belong to three fungal groups in the genus *Mycena* (Fig. 1b; Ogura-Tsujita et al. 2009). By contrast, *Gastrodia pubilabiata*, a species closely related to *G. confusa*, associates with multiple groups of litter-decaying fungi in the families Mycenaceae, Marasmiaceae, and Omphalotaceae (Fig. S2; Kinoshita et al. 2016). Further, a close relative of these *Gastrodia* species, *Gastrodia nipponica*, associates with wood-decaying and ECM fungi in addition to litter-decaying fungi, and its mycobionts exhibited significantly higher sequence divergence than those of *G. confusa* and *G. pubilabiata* (Kinoshita et al. 2016). The giant mycoheterotroph, *Erythrorchis altissima*, is an extreme example of low fungal specificity in full MHPs. In total, 37 fungal species belonging to nine orders of Basidiomycota, which mainly include wood-decaying fungi but also ECM and rhizoctonia fungi, have been identified in the roots of this MHP (Ogura-Tsujita et al. 2018). Mycobiont specificity in SAP-MHPs often varies within a single host plant genus, as in *Gastrodia* (Kinoshita et al. 2016) and *Yoania* (Yamashita et al. 2020). Therefore, specificity can fluctuate greatly during host-plant speciation.

## Fungal partner shift during the plant life cycle

The fungal partner often changes during the life cycle of an SAP-MHP. A mycoheterotrophic orchid, *Gastrodia elata*, switches its fungal partner upon transitioning from the juvenile to adult stage. The litter-decaying fungus, *Mycena*, induces seed germination, whereas the wood-decaying *Armillaria* supports further development of the mature plant (Xu and Guo 2000). A recent high-throughput sequencing study suggests that more diverse fungal groups than previously assumed are involved at the juvenile stage of *G. elata* (Chen et al. 2019). Partner switching also seems to occur in *Cyrtosia septentrionalis*, the adult stage of which is associated with *Armillaria* (e.g., Hamada 1939). However, *C. septentrionalis* seeds failed to germinate with *Armillaria* isolates *in vitro* (Terashita 1985), and *Physisporinus*, a wood-decaying fungus, promoted germination *in situ* (Umata et al. 2013). Changing the fungal partner at some stage of the life cycle seems riskier than living with the same partner. Switching to a fungus with a large biomass, such as *Armillaria*, allows access to a large carbon pool, thus possibly outweighing the risk of partner shifting. *Armillaria* produces abundant rhizomorphs in soil (Smith et al. 1992), which increases the likelihood of successful colonization.

## Evolution of mycorrhizal interactions

During the evolution from autotrophy to mycoheterotrophy, the associated mycorrhizal fungi have switched to different fungal communities in some instances (Jacquemyn and Merckx 2019; Ogura-Tsujita et al. 2012; Yagame et al. 2016). Most leafy relatives of SAP-MHPs are associated with rhizoctonia fungi, suggesting that the mycorrhizal community shifted from rhizoctonia to litter- or wood-decaying fungi during the evolution of SAP-MHP lineages. The chlorophyllous genus *Vanilla* is most closely related to three genera containing SAP-MHPs in the tribe Vanilleae (Cameron 2011; Cameron et al. 2009; Fig. S1) and mainly associates with rhizoctonia fungi, including Tulasnellaceae and Ceratobasidiaceae (Porras-Alfaro and Bayman 2007). Mycobionts may have shifted from rhizoctonia to diverse wood-decaying fungi in accordance with the evolution of *Galeola-Cyrtosia* and *Erythorchis*. The leafy genus *Calypso* is likely sister to SAP-MHP-containing *Yoania* (Freudenstein et al. 2017) and forms associations with Tulasnellaceae and Ceratobasidiaceae (Currah et al. 1988; Taylor and McCormick 2008). This suggests that fungal partners have been switched from rhizoctonia fungi to the wood-decaying *Physisporinus* with the shift to SAP-MH in *Yoania*. Rhizoctonia fungi are often found as free-living saprotrophs (Roberts 1999), and mycorrhizal associations with these fungi are unique to Orchidaceae (Yukawa et al. 2009). This ability to associate with free-living fungi in orchids might have triggered the evolution of full mycoheterotrophy dependent on saprotrophic non-rhizoctonia fungi.

Mycorrhizal communities are typically switched via a phase of dual association, which involves mycobionts of both leafy and leafless plants, during the evolution of mycoheterotrophy. In the orchid genus *Cymbidium*, mycobionts were compared within a clade in which mycoheterotrophy evolved (Ogura-Tsujita et al. 2012). A leafy outgroup species, *Cymbidium dayanum* Rchb.f., associates mainly with rhizoctonia fungi from Tulasnellaceae, whereas two MHPs, *Cymbidium macrorhizon* Lindl. and *Cymbidium aberrans* (Finet) Schltr., mostly associate with ECM fungi in Sebacinaceae. By contrast, the leafy sister taxa of the two MHPs, *Cymbidium lancifolium* Hook. and *Cymbidium goeringii* (Rchb.f.) Rchb.f., associate with both rhizoctonia fungi and several ECM fungal families, suggesting that fungal partners have switched via a dual association with both rhizoctonia and ECM fungi. Such dual associations could trigger mycorrhizal switching and play an important role in the evolution of MHPs. Another case of evolution via a dual association with wood-decaying fungi was found in the genus *Cremastra* (Yagame et al. 2018). A SAP-MHP, *Cremastra aphylla*, which mainly associates with *Coprinellus*, a wood-decaying fungus, and its leafy sister species, *Cremastra appendiculata* (D.Don) Makino, can associate with both rhizoctonia fungi and *Coprinellus* (Freudenstein et al. 2017; Nishikawa and Ui 1976; Yagame et al. 2013). This suggests that the type of mycorrhizal fungi in the symbiosis had been switched to wood-decaying fungi via a dual association. A novel association with *Coprinellus* probably triggered the evolution of the SAP-MHP.

Symbiotic associations with SAP fungi are occasionally found in leafy orchid species. The wood-decaying fungus *Psathyrella* is often found associating with leafy orchids, such as *Oeceoclades maculata* (Lindl.) Lindl. (Bayman et al. 2016) and *Satyrium nepalense* D.Don (Jyothsna and Purushothama 2014). Seeds of the former orchid species germinated *in vitro* only in association with *Psathyrella*, whereas adult plants associate with rhizoctonia fungi in addition to *Psathyrella* (Bayman et al. 2016). Such high specificity for SAP fungi during seed germination could represent an initial stage of the evolution of an SAP-MHP. *Mycena* fungi are one of the main symbionts of *Gastrodia* SAP-MHPs and also promote seed germination and seedling growth in *Dendrobium* epiphytic green orchids (Guo et al. 1997; Zhang et al. 2012). Interestingly, *Mycena anoectochila* L. Fan & S.X. Guo, which was isolated from the leafy orchid *Anoectochilus roxburghii* (Wall.) Lindl., induced seed germination in the mycoheterotrophic *Gastrodia elata* (Guo et al. 1997). These results suggest that the same fungus can associate with both leafy and leafless orchids. *Mycena* fungi were also found to associate with the terrestrial orchids *Cymbidium sinense* (Andrews) Willd. (Fan et al. 1996), *Goodyera repens* (L.) R.Br. (Voronina et al. 2018), and *Bletilla striata* (Thunb.) Rchb.f. (Guo and Xu 1992). Members of Trechisporales, Polyporales, Corticiales, and Hymenochaetales, which largely comprise wood-decaying fungi, have been occasionally found in tropical epiphytic orchids (Kartzinel et al. 2013; Martos et al. 2012). Although these cases could reflect opportunistic associations in leafy orchids, a symbiotic relationship with SAP fungi may be more adaptive than that with rhizoctonia fungi in some environments and trigger the evolution of SAP-MHPs (Selosse et al. 2010).

Convergent evolution of mycorrhizal specificity toward particular fungal lineages has occurred in several SAP-MH lineages. Associations with *Armillaria* have been observed for *Cyrtosia septentrionalis* (Subfamily Vanilloideae) and *Gastrodia elata* (Subfamily Epidendroideae) (Fig. 2). For instance, *Armillaria gallica* associates with both these phylogenetically distant orchids exhibiting SAP-MH (Kikuchi et al. 2008a; Terashita 1996). Two SAP-MHPs among several tribes of Epidendroideae, *Epipogium roseum* and *Eulophia zollingeri*, associate with Psathyrellaceae fungi (Ogura-Tsujita and Yukawa 2008; Yamato et al. 2005). Sequences of the nuclear ribosomal ITS region of *Psathyrella* fungi isolated from these two orchids had > 97% similarity (Ogura-Tsujita and Yukawa 2008). Such convergent evolution has also been found in AM- and ECM-MHPs. AM fungi from AM-MHPs belonging to different plant families, such as Burmanniaceae and Corsiaceae, were grouped into the same taxa (> 97% small subunit rRNA sequence similarity; Gomes et al. 2019; Merckx et al. 2012). The mycobionts in Sebacinaceae from the ECM-MHP *Hexalectris* (Epidendreae) are closely related to those from *Neottia nidus-avis* (L.) Rich. (Neottieae) (> 98% ITS sequence similarity; Kennedy et al. 2011). Mycobionts of MHPs may converge on particular fungal taxa that support high nutrient acquisition.

Mycorrhizal interactions have changed during the speciation of MHPs. The species of mycobionts in the associations have changed or fungal specificity has changed from broad to narrow, such that the same mycobiont species is shared among several plant species (Kinoshita et al. 2016). In SAP-MHPs, fungal specificity differed among three closely related *Gastrodia* species, *G. confusa*, *G. pubilabiata*, and *G. nipponica*, all of which are associated with litter-decaying fungi within Mycenaceae, Marasmiaceae, and Omphalotaceae (Kinoshita et al. 2016). The fungal specificity of *G. confusa* was significantly greater than that of *G. pubilabiata* and *G. nipponica*, indicating that specificity fluctuates during speciation. Interestingly, *G. confusa* exclusively inhabits bamboo thickets, the mycorrhizal communities of which differ significantly from those of other vegetation. This suggests that adaptation to particular fungi inhabiting bamboo thickets triggered the speciation of *G. confusa*. The mycobionts of 15 *Gastrodia* species have been surveyed; Mycenaceae, Marasmiaceae or Omphalotaceae fungi were reported to associate with ten species (Table 1). This suggests that these litter-decaying fungi are main fungal partners for *Gastrodia* species. Associations with the wood-decaying fungi *Armillaria* and *Resinicium* could have appeared during the evolution of *Gastrodia elata* and *Gastrodia simillis*. Furthermore, a symbiotic relationship with ECM fungi has been observed in *G. nipponica*, which associates with both litter-decaying and ECM fungi. Both SAP- and ECM-MHPs have appeared in the genus *Epipogium* (Roy et al. 2009; Yamato et al. 2005). Although because of the poor phylogenetic resolution of this genus it is unclear which MHP type evolved earlier, the fungal partner could be switched to SAP or ECM fungi even in closely related taxa. Fungal partner switching during speciation has been reported in AM- and ECM-MHPs. Two sister ECM-MHPs, *Corallorhiza maculata* (Raf.) Raf. and *Corallorhiza mertensiana* Bong., specifically associate with different Russulaceae fungal taxa (Taylor and Bruns 1999). Fungal specificity differs among the six ECM-MHPs within *Neottia* (Yagame et al. 2016). Five AM-MHPs in the genus *Afrothismia* exhibit high fungal specificity for different Glomeraceae fungal taxa (Merckx and Bidartondo 2008; Merckx et al. 2012). These results suggest that switching between mycorrhizal partners accelerated the speciation of MHPs.

## Isotopic signature of SAP-MHPs

Nutrient fluxes between MHPs and mycorrhizal fungi have been studied using stable isotope natural abundance analysis (Gebauer and Meyer 2003; Hynson et al. 2013). Because fungal-derived carbon and nitrogen are highly enriched in ^13^C and ^15^N, the tissues of MHPs are expected to also be enriched in carbon and nitrogen isotopes compared to the surrounding autotrophic plants (Gebauer and Meyer 2003). This approach has been applied to examine a variety of ECM-MHPs (Liebel and Gebauer 2011; Motomura et al. 2010; Roy et al. 2009), AM-MHPs (County et al. 2011; Gomes et al. 2020; Merckx et al. 2010), and several SAP-MHPs (Lee et al. 2015; Martos et al. 2009; Ogura-Tsujita et al. 2009). The relative enrichment levels of isotopes confirmed the mycoheterotrophy of those species. All three types of MHPs are highly enriched in ^13^C, but the ^15^N enrichment level is lower in SAP- and AM-MHPs than in ECM-MHPs. The difference is attributable to the greater enrichment of nitrogen isotopes in ECM fungi than in AM and SAP fungi, as a result of their different nitrogen acquisition strategies. Interestingly, litter-decaying fungi are generally depleted in carbon isotopes relative to wood-decaying fungi (Kohzu et al. 1999), likely because wood is more enriched in ^13^C than leaf tissue (Gebauer and Schulze 1991). Lee et al. (2015) showed that ^13^C was significantly less enriched in SAP-MHPs associated with the litter-decaying fungi—i.e., *Gastrodia appendiculata* C.S.Leou & N.J.Chung, *Gastrodia fontinalis* T.P.Lin, and *Gastrodia nantoensis* T.C.Hsu & C.M.Kuo ex T.P.Lin—than in those associated with wood-decaying fungi. However, there are insufficient studies of the isotopic signature of SAP-MHPs, and further work is required to clarify the isotopic signatures of litter- and wood-decaying fungi and elucidate the physiological ecology of SAP-MHPs.

Stable isotope analysis can be used to distinguish between partial and full mycoheterotrophy (Gebauer and Meyer 2003). The linear two-source mixing model estimates the proportion of carbon and nitrogen gain from photosynthesis and mycorrhizal fungi. The endpoint of this model is a value that falls between those of co-occurring autotrophic plants (0% nutrient gain from fungi) and full MHPs (100% nutrient gain from fungi) (Preiss and Gebauer 2008). The level of mycoheterotrophy has been quantitatively assessed for various leafy plant species associated with ECM (Abadie et al. 2006; Bidartondo et al. 2004; Matsuda et al. 2012) and AM (Suetsugu et al. 2020a) fungi using this model, but little is known of those associated with SAP fungi. A preliminary isotope analysis by Yagame et al. (2015) showed that the leafy orchid *Cremastra appendiculata*, which associates with wood-decaying fungi, is partially mycoheterotrophic. Stable isotope analysis may reveal more diverse partial SAP-MHPs if applied to leafy orchids associated with non-rhizoctonia SAP fungi.

Suetsugu et al. (2020b) estimated the age of carbon in SAP-MHP tissue by measuring the natural abundance of radiocarbon (nuclear weapon-derived ^14^C). This approach traces the time elapsed since carbon isotopes derived from the nuclear-weapon tests of the 1950s and 1960s were fixed from atmospheric CO_2_ by photosynthesis. The carbon utilized by wood decaying fungus-dependent MHPs was fixed 10–40 years before that fixed by ECM-MHPs (Suetsugu et al. 2020b). The carbon in SAP-MHPs associated with litter-decaying fungi was estimated to be 6.7–9.9 years old (Hatté et al. 2020), suggesting that SAP-MHPs associated with wood-decaying fungi use older carbon than those associated with litter-decaying fungi. This technique will enable investigations of nutrient flows via mycoheterotrophy from decomposing plant debris.

## Culturing SAP-MHPs

AM and ECM fungi are almost obligately biotrophic, i.e., dependent on autotrophic plants for their carbon supply. Thus, AM- and ECM-MHPs require a chlorophyllous host plant for co-culture with appropriate symbiotic fungi *in vitro* (Mckendrick et al. 2000). By contrast, mycobionts of SAP-MHPs can grow in pure culture and stimulate seed germination and further seedling growth *in vitro* (Burgeff 1932; Yagame et al. 2007). Field or container culture has been established for several SAP-MHPs (Shimaoka et al. 2017; Xu and Guo 2000). These culture techniques will enhance our understanding of the physiology of mycoheterotrophy.

*In vitro* symbiotic culture with MHP seeds and their mycobiont was achieved for SAP-MHPs by Burgeff (1932). Leaf litter or wood debris is the main carbon source for fungal isolates from SAP-MHPs, and so culture medium containing dead organic material, such as fallen leaves, twigs, or ground wood chips, can sustain isolates. Pieces of *Quercus* leaves inoculated with *Mycena osmundicola* J.E. Lange induced seed germination in *Gastrodia elata* on water agar without additives (Kim et al. 2006). In a preliminary study, we induced germination of *Gastrodia nipponica* using this system (Fig. 1i). A medium containing *Fagus crenata* Blume sawdust, water, glucose, and yeast powder enabled co-culture of seeds of *Erythrorchis altissima* and fungi in a series of studies by Umata (1995, 1997a, b, 1998a, b, 1999) (Fig. 1h; Table 1). The seedlings continued to grow after they were transplanted into large culture bottles with fresh sawdust medium (Fig. 1h), attained a stem length of more than 30 cm, and were successfully transplanted to their natural habitat (Umata et al. 2007). Symbiotic culture in a medium containing bamboo leaves and nutrient solution induced flower bud formation in *Gastrodia verrucosa* Blume (Tashima et al. 1978). Mycobionts of the mycoheterotrophic orchids *Galeola nudifolia* Lour. (previously known as *Galeola hydra* Rchb.f.) and *Gastrodia javanica* Endl. grew on medium containing pure cellulose or lignin from *Populus* wood (Burgeff 1936; Hollander 1932); thus, these components may be crucial for the growth of SAP-MHPs.

Artificial cultivation other than *in vitro* culture, such as in the field or in a container, has been established for several SAP-MHPs. *Gastrodia elata*, a component of a traditional Chinese medicine, has been cultivated in the field using wood logs inoculated with *Armillaria mellea* (Park and Lee 2013a; Xu and Guo 2000). The development of this cultivation technique has been useful to the pharmaceutical industry. Flower induction under symbiotic cultivation in a container was achieved for *Epipogium roseum* using sawdust and volcanic soil (Yagame et al. 2007). Container cultivation of *Gastrodia* species has been achieved using organic materials from their natural habitats. Seed germination in *Gastrodia nipponica* (Umata and Nishi 2010) and *Gastrodia pubilabiata* (Higaki et al. 2017) was induced in a plastic box containing leaf litter from their habitats. Further, the life cycles of *G. pubilabiata* and *Gastrodia confusa* were completed in culture with wood logs, cedar cones, and humus from a forest (Shimaoka et al. 2017).

Asymbiotic culture is difficult for full MHPs, but several studies have demonstrated *in vitro* asymbiotic germination and subsequent growth of SAP-MHPs. Controlling the O_2_ and CO_2_ concentrations within the culture vessel stimulates seed germination in *Cyrtosia septentrionalis* (Nakamura et al. 1975). Interestingly, the concentrations were similar to those in soil, implying that in its natural habitat, the seed does not require direct mycobiont contact for germination (Umata et al. 2013). Aseptically germinated seedlings developed inflorescences in *Didymoplexis pallens* (Irawati 2002), and rhizome formation was observed in an asymbiotic culture of *Yoania flava* K.Inoue & T.Yukawa (Tsuda et al. 2004). Aseptic propagation via an embryogenic callus was demonstrated in *Gastrodia elata*, and the regenerated tubers continued to grow after inoculation of *Armillaria* isolates (Yeh et al. 2017). Seeds of *Gastrodia pubilabiata* successfully germinated without symbionts, and their subsequent development was controlled by illumination (Godo et al. 2020).

## Future perspectives

Fully mycoheterotrophic plants associated with SAP fungi have to date been found only in Orchidaceae and have evolved independently at least nine times within two subfamilies, Vanillioideae and Epidendroideae. A variety of litter- and wood-decaying fungi are involved in mycoheterotrophy in association with SAP-MHPs, and several SAP-MHPs can be cultured with or without mycorrhizal fungi. Culturable SAP-MHPs may be key to addressing many unsolved questions regarding mycoheterotrophy and will contribute to a range of scientific fields. A critical event in the evolution from autotrophy to mycoheterotrophy is fungal partner switching, the replacement of the associated fungal community by another. The mycobionts of most SAP-MHPs have been switched from rhizoctonia fungi to leaf-litter- or wood-decaying basidiomycetes. The benefits gained by plants from mycobionts differ between rhizoctonia and SAP fungi. Plants may select the best fungal partners for nutrient acquisition (Jacquemyn and Merckx 2019; Ogura-Tsujita et al. 2012), thus triggering the evolution of mycoheterotrophy. Symbiotic culture will allow direct comparisons of the relative fitness between plants with rhizoctonia and those with SAP mycobionts. Leafy sister species of fully mycoheterotrophic species, such as *Cremastra appendiculata*, often associate with rhizoctionia and wood-decaying fungi and could be suitable model plants for such assays. Comparing gene expression between SAP-MHPs and their leafy relatives will clarify the mechanism underlying the evolution from autotrophy to mycoheterotrophy. Field samples are subject to environmental effects, but culture systems facilitate comparisons between autotrophs and mycoheterotrophs.

The physiological mechanisms underlying plant–fungus interactions in MHPs are unclear, but recent studies of SAP-MHPs have provided information on the interactions between MHPs and their mycorrhizal fungi. Transcriptomic and proteomic analyses of *Gastrodia elata* co-cultured with *Mycena* fungi revealed differentially accumulated mRNAs and proteins involved in energy metabolism, plant defense, molecular signaling, and secondary metabolism (Zeng et al. 2017, 2018). Fungal digestion was demonstrated during seed germination in *G. elata* co-cultured with *Mycena* (Li et al. 2020). The factors transported from the fungus to the plant are unknown in MHPs, but two sucrose transporter-like genes, *GeSUT4* and *GeSUT3*, were highly expressed in *Armillaria*‐colonized *G. elata* tubers, suggesting that sucrose is the major sugar transported between the fungus and *G. elata* (Ho et al. 2020). Symbiotic culture of SAP-MHPs enables broader approaches for physiological studies of mycoheterotrophy, such as those investigating the mechanism of recognition between plant and fungus, and of nutrient transfer from fungus to plant. Furthermore, asymbiotic culture allows the comparison of gene expression profiles of plants with and without mycorrhizal fungi, thus providing insights into the physiology of mycoheterotrophy.

The ecology of MHPs is poorly understood because they spend most of their life cycle underground and shoot systems appear only during reproductive phases. For example, the processes of plant development and seasonal growth in many MH species are considered “black boxes”. A culture system would expand the understanding of the life cycle and phenological properties of MHPs. Container culture of *Epipogium roseum* revealed the developmental process of subterranean seedlings, with stolons and rhizomes produced (Yagame et al. 2007). Rapid clonal propagation of this orchid was also achieved, with a single protocorm producing 80 tubers. Development from seed to flower in several SAP-MHPs was successfully monitored in symbiotic or asymbiotic culture and required 6 months in *E. roseum* (Yagame et al. 2007), 4 months in *Gastrodia pubilabiata* (Shimaoka et al. 2017), and 4–6 months in *Didymoplexis pallens* (Irawati 2002). Many MHPs are endangered worldwide because of habitat loss and climate change (Merckx 2013). A culture system would contribute to the recovery of the SAP-MHP populations. The rhizomes of *Yoania flava* that developed under symbiotic culture were transplanted to the natural habitat and survived for 490 days thereafter (Tsuda et al. 2004). Because MHPs require mycobionts for survival, *ex situ* conservation of plants with their mycobionts is a good strategy for preventing extinction. The cryopreservation of seeds and culture of SAP-MHP symbiont isolates will contribute greatly toward the long-term conservation of SAP-MHPs.

## Supplementary Information

Below is the link to the electronic supplementary material.

Supplementary file1 (PDF 395 KB)
