# Supplementary material for: Evolutionary histories and mycorrhizal associations of mycoheterotrophic plants dependent on saprotrophic fungi
Source: J Plant Res. 2021 Jan 8;134(1):19–41. doi: 10.1007/s10265-020-01244-6 (PMC7817554; doi:10.1007/s10265-020-01244-6)
Supplement: Supplementary file 1 — Supplementary file1 (PDF 395 KB) [file 10265_2020_1244_MOESM1_ESM.pdf]

Electronic Supplementary Material

Evolutionary histories and mycorrhizal associations of mycoheterotrophic plants dependent on saprotrophic fungi

Journal of Plant Research

Ogura-Tsujita Y, Yukawa T, Kinoshita A

Corresponding author: Ogura-Tsujita Y
Faculty of Agriculture, Saga University, 1 Honjyo-machi, Saga 840-8752, Japan
ytsujita@cc.saga-u.ac.jp

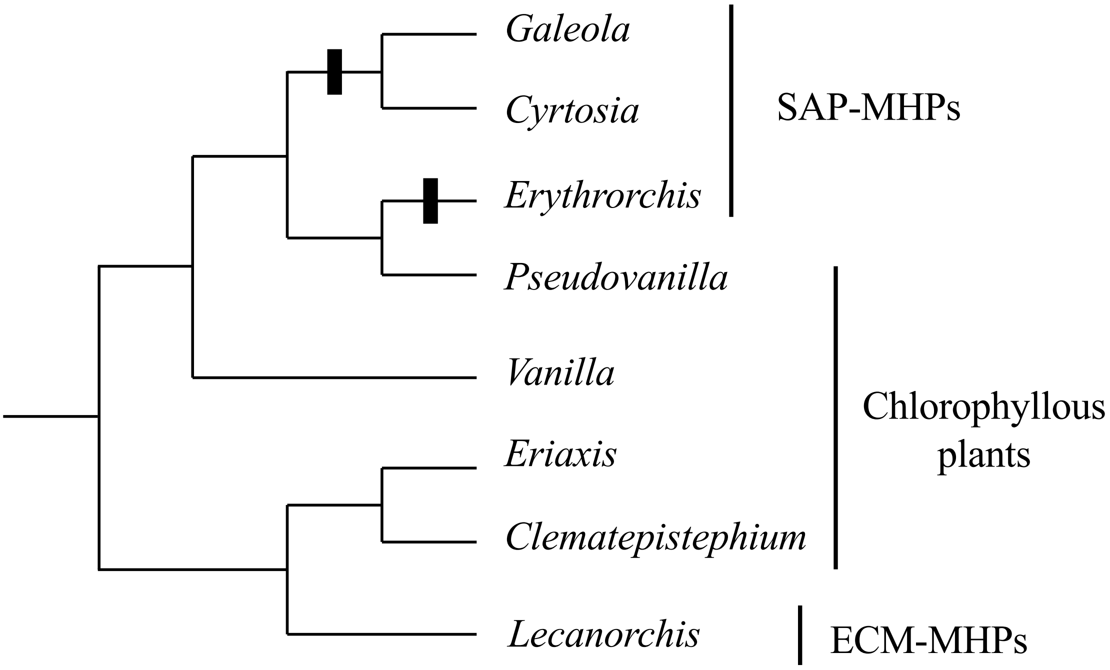

Fig. S1 Evolutionary course of saprophytic fungi-associated mycoheterotrophic plants (SAP-MHPs) in subfamily Vanilloideae, Orchidaceae. Bars show points where SAP-MHPs likely evolved. The phylogram of Vanilloideae is summarized from Cameron et al. (2009) and Cameron (2011).

Fig. S2 Phylogenetic placements of saprophytic fungi-associated mycoheterotrophic plants (SAP-MHPs) within Marasmiaceae and Omphalotaceae as inferred from maximum likelihood (ML) analysis based on ribosomal DNA internal transcribed spacer sequences. After multiple alignment of 34 SAP-MHP sequences with 204 published sequences of Omphalotaceae and Marasmiaceae using MAFFT7 (Katoh and Standley 2013), ML analysis was conducted using the IQ-TREE web server (Trifinopoulos et al. 2016) with SH-like-aLRT branch support and a best fit GTR+F+R5 evolutionary model as selected by ModelFinder (Kalyaanamoorthy et al. 2017). Clade name assignment for Marasmiaceae and Omphalotaceae was in accordance with Antonín et al. (2019), Oliveira et al. (2019), and Sandoval-Leiva et al. (2016). Mycobionts of SAP-MHPs are indicated in bold and clades without SAP-MHPs mycobionts are collapsed. SH-aLRT values ≥ 75% are shown below the branches.

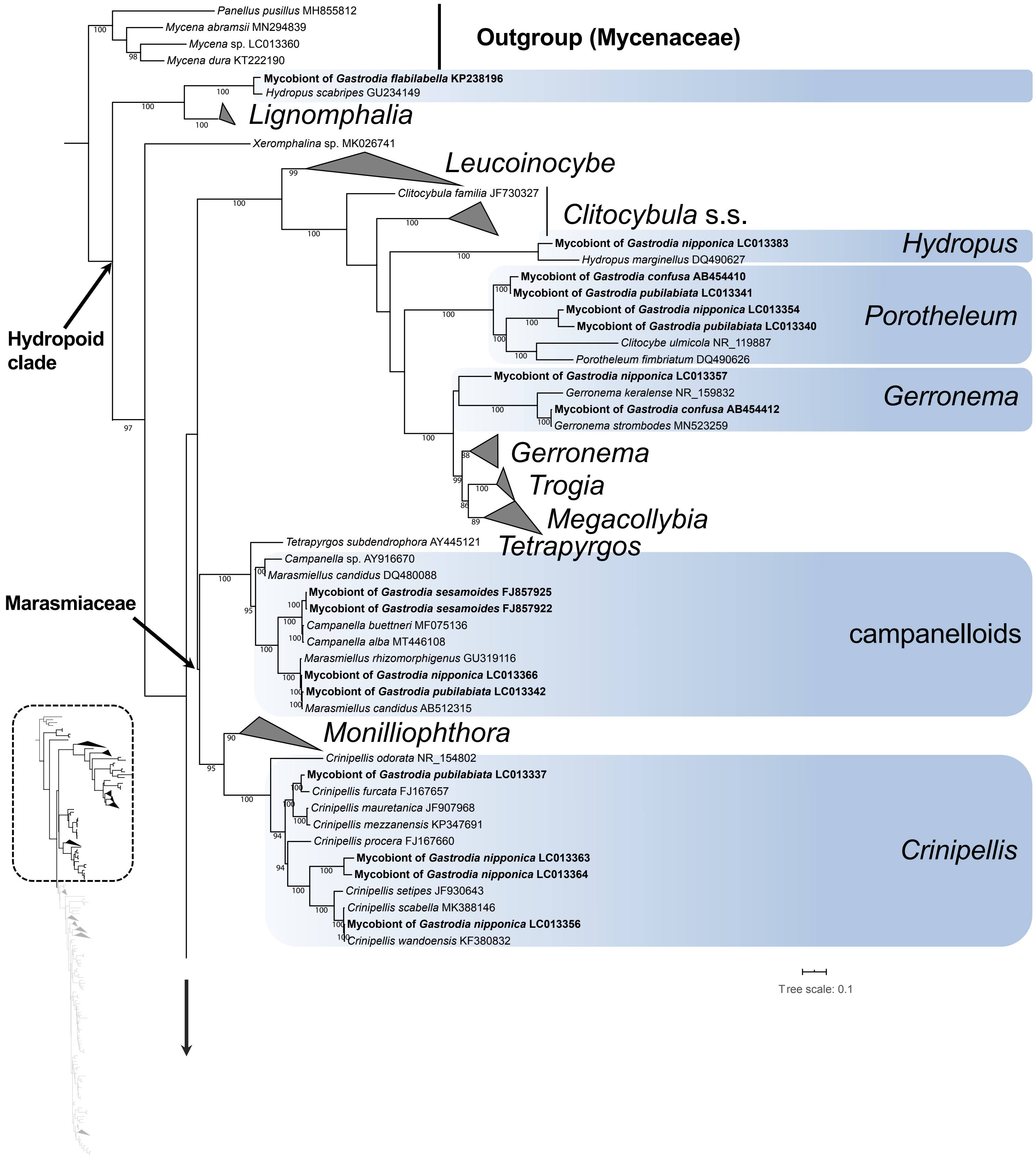

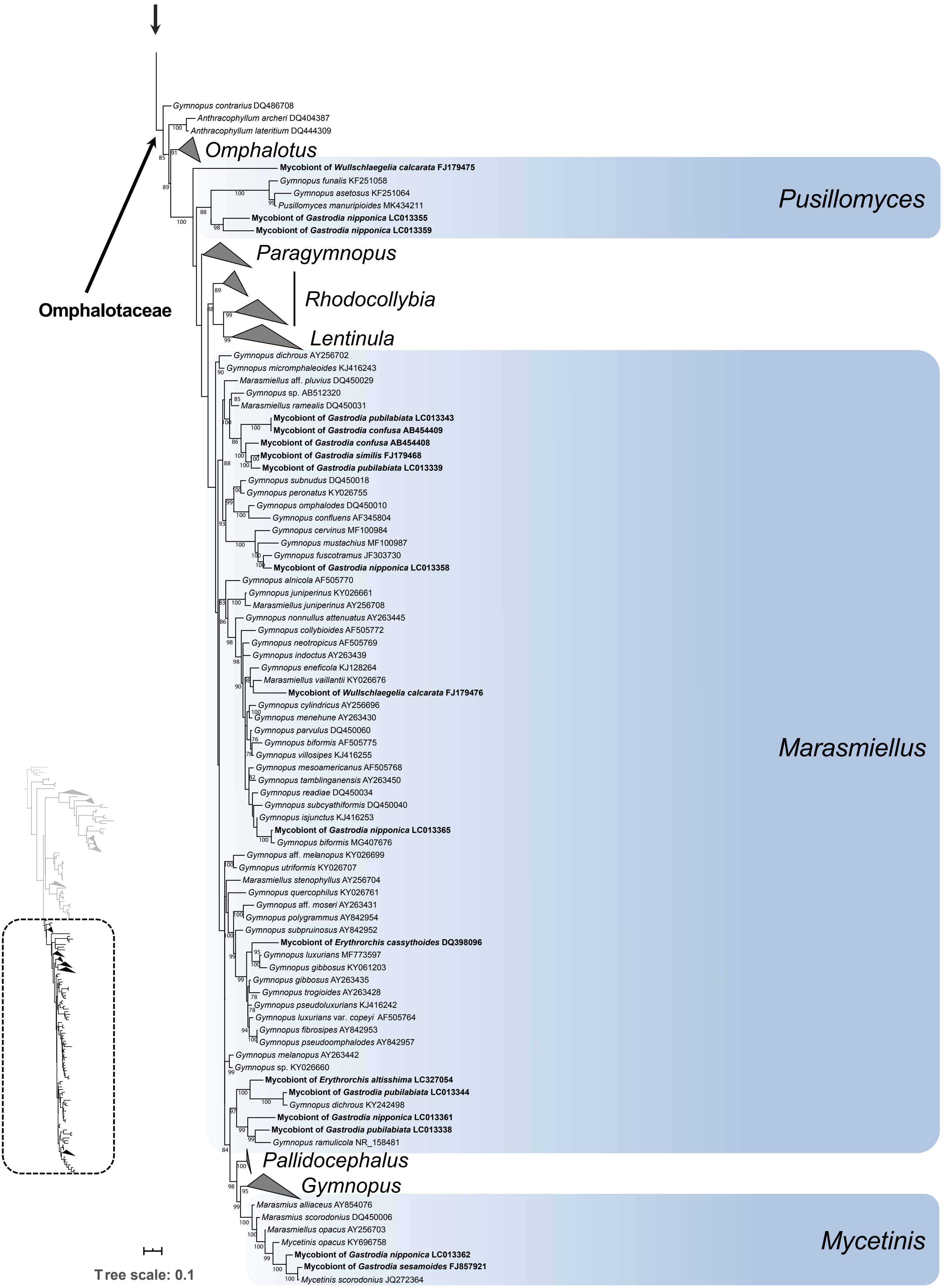
Omphalotaceae
Gymnopus contrarius DQ486708
Anthracophyllum archeri DQ404387
Anthracophyllum lateritium DQ444309
Omphalotus
Mycobiont of Wullschlaegelia calcarata FJ179475
Gymnopus funalis KF251058
Gymnopus asetosus KF251064
Pusillomyces manuripioides MK434211
Mycobiont of Gastrodia nipponica LC013355
Mycobiont of Gastrodia nipponica LC013359
Pusillomyces
Paragymnopus
Rhodocollybia
Lentinula
Gymnopus dichrous AY256702
Gymnopus micromphaleoides KJ416243
Marasmiellus aff. pluvius DQ450029
Gymnopus sp. AB512320
Marasmiellus ramealis DQ450031
Mycobiont of Gastrodia pubilabiata LC013343
Mycobiont of Gastrodia confusa AB454409
Mycobiont of Gastrodia confusa AB454408
Mycobiont of Gastrodia similis FJ179468
Mycobiont of Gastrodia pubilabiata LC013339
Gymnopus subnudus DQ450018
Gymnopus peronatus KY026755
Gymnopus omphalodes DQ450010
Gymnopus confluens AF345804
Gymnopus cervinus MF100984
Gymnopus mustachius MF100987
Gymnopus fuscotramus JF303730
Mycobiont of Gastrodia nipponica LC013358
Gymnopus alnicola AF505770
Gymnopus juniperinus KY026661
Marasmiellus juniperinus AY256708
Gymnopus nonnullus attenuatus AY263445
Gymnopus collybioides AF505772
Gymnopus neotropicus AF505769
Gymnopus indoctus AY263439
Gymnopus eneficola KJ128264
Marasmiellus vaillantii KY026676
Mycobiont of Wullschlaegelia calcarata FJ179476
Gymnopus cylindricus AY256696
Gymnopus menehune AY263430
Gymnopus parvulus DQ450060
Gymnopus biformis AF505775
Gymnopus villosipes KJ416255
Gymnopus mesoamericanus AF505768
Gymnopus tamblinganensis AY263450
Gymnopus readiae DQ450034
Gymnopus subcyathiformis DQ450040
Gymnopus isjunctus KJ416253
Mycobiont of Gastrodia nipponica LC013365
Gymnopus biformis MG407676
Gymnopus aff. melanopus KY026699
Gymnopus utriformis KY026707
Marasmiellus stenophyllus AY256704
Gymnopus quercophilus KY026761
Gymnopus aff. moseri AY263431
Gymnopus polygrammus AY842954
Gymnopus subpruinosus AY842952
Mycobiont of Erythrorchis cassythoides DQ398096
Gymnopus luxurians MF773597
Gymnopus gibbosus KY061203
Gymnopus gibbosus AY263435
Gymnopus trogioides AY263428
Gymnopus pseudoluxurians KJ416242
Gymnopus luxurians var. copeyi AF505764
Gymnopus fibrosipes AY842953
Gymnopus pseudoomphalodes AY842957
Gymnopus melanopus AY263442
Gymnopus sp. KY026660
Mycobiont of Erythrorchis altissshima LC327054
Mycobiont of Gastrodia pubilabiata LC013344
Gymnopus dichrous KY242498
Mycobiont of Gastrodia nipponica LC013361
Mycobiont of Gastrodia pubilabiata LC013338
Gymnopus ramulicola NR_158481
Marasmiellus
Pallidocephalus
Gymnopus
Marasmius alliaceus AY854076
Marasmius scorodonius DQ450006
Marasmiellus opacus AY256703
Mycetinis opacus KY696758
Mycobiont of Gastrodia nipponica LC013362
Mycobiont of Gastrodia sesamoides FJ857921
Mycetinis scorodonius JQ272364
Mycetinis
Tree scale: 0.1
